# Cryptochrome-mediated blue light regulates cell lignification via PbbHLH195 activation of the PbNSC in pear fruits

**DOI:** 10.1186/s43897-025-00149-z

**Published:** 2025-05-07

**Authors:** Qi Wang, Xinyi Wu, Mei Ren, Fanghang Zhang, Yang Zhang, Yueyang Wang, Wen Li, Zhihua Xie, Kaijie Qi, Shaoling Zhang, Katsuhiro Shiratake, Yingying Niu, Shutian Tao

**Affiliations:** 1 Sanya Institute, State Key Laboratory of Crop Genetics and Germplasm Enhancement, College of Horticulture, Nanjing Agricultural University, Nanjing, 210095 China; 2 Laboratory of Horticultural Science, Graduate School of Bioagricultural Sciences, Nagoya University, Nagoya, 464-8601 Japan; 3 College of Horticulture, Xinjiang Agricultural University, Urumqi, 830052 China; 4 College of Horticulture, Anhui Agricultural University, Hefei, 230036 China

**Keywords:** Pear, Blue light, Lignification, Stone cell, Regulating module

## Abstract

The presence of stone cells in pear fruit, caused by lignified secondary cell walls (SCWs), leads to a grainy texture in the fruit flesh, thereby compromising its overall quality. Lignification is influenced by various environmental signals, including light, however the underlying mechanism are poorly understood. This study reveals that SCW thickening and lignin accumulation in stone cells were regulated by a blue light signal, mediated through the activation of *PbNSC* by* PbbHLH195*. The results revealed that the stone cell formation was prompted by supplementary with blue light, with lignin accumulation linked to the upregulation of the *NAC STONE CELL PROMOTING FACTOR* (*PbNSC*). *PbbHLH195* was identified as a novel molecular hub connecting lignification to blue light signal through its physical interaction with PbCRY1a. The biochemical and functional analysis indicates that PbbHLH195 contributes to stone cell lignification by activating the promoter of *PbNSC*. Our findings offer novel insights into the mechanisms of lignin biosynthesis in response to blue light, identifying valuable genetic targets for enhancing the fruit quality of pear.

**Supplementary Information:**

The online version contains supplementary material available at 10.1186/s43897-025-00149-z.

## Core

Lignin accumulation in pear fruit stone cells was regulated by a blue light signal that is mediated via *PbbHLH195* activation of *PbNSC. *PbbHLH195 was identified as a novel molecular hub connecting lignification with blue light signal, and interacts with PbCRY1a.

## Gene & Accession Numbers

Information of genes in this study can be found in the database (http://peargenome.njau.edu.cn/) under the accession numbers: *PbCRY1a* (*Pbr024556.1*); *PbbHLH195 *(*Pbr042466.1*); *PbNSC* (*Pbr038584.1*).

## Introduction

Lignin, is deposited in the secondary cell wall (SCW) of all vascular plants and represents approximately 30% of the organic carbon in the biosphere (Boerjan et al. 2003). The formation of thickened SCWs and lignin production provides plants with the mechanical strength required for upright growth and the ability to conduct water and nutrients over long distances (Somerville et al. 2010).This lignification process also occurs in certain fruits, significantly influencing their quality. such as pear (Tao et al. 2009), loquat (Shan et al. 2008), mangosteen (Bunsiri et al. 2003), pummelo (Shi et al. 2020), zucchini, and kiwifruit (Suo et al. 2018).

Stone cells of pear fruit are a type of sclerenchyma cells formed through the thickening of SCWs and the lignin deposition on the primary walls of parenchyma cells (Smith 1935; Tao et al. 2009). Similar to lignin found in most angiosperms, the lignin composition in pear stone cells consists of guaiacyl lignin (G-lignin), with smaller amounts of p-hydroxyphenyl lignin (H-lignin) and syringyl lignin (S-lignin) (Jin et al. 2013; Yan et al. 2014). The lignification in plants is strongly influenced by various environmental factors, mediated through receptors or transcriptional hubs that activate critical developmental pathways. Among these factors, light plays a pivotal role (Hu et al. 2021; Luo et al. 2022; Rogers et al. 2005), but the underlying regulatory mechanisms are still limited. Understanding the molecular hubs that connect light signals with developmental signals can enable us to regulate lignification effectively and align it with plant development and environmental adaptation.

Light is an essential resource for plants, profoundly influencing their physiology and development by regulating various processes, from photosynthesis to secondary metabolism, through the activation of receptors or transcriptional hubs that control first-layer developmental switches (Lazzarin et al. 2021). Changes in light conditions, particularly in spectral composition, have been associated with lignification across different developmental processes and species (Syros et al. 2005). For instance, light induces lignin deposition in root of Arabidopsis (Hemm et al. 2004), and the transcript levels of genes encoding lignin biosynthetic enzymes exhibit circadian oscillation (Rogers et al. 2005). Phenylalanine ammonia-lyase (PAL), encoding the first enzyme in lignin biosynthesis was induced by blue light and ultraviolet (Bufler and Bangerth 1982; Engelsma 1974). The blue light receptor, CRYPTOCHROME1 (CRY1) is crucial for photomorphogenesis (Ahmad and Cashmore 1993), and has been shown to be involved in SCW thickening (Zhang et al. 2018) and programmed cell death (Danon et al. 2006). In *Atcry1* mutants, multiple genes related to lignin biosynthesis and regulation, including key switches like *SND1*, *SND2*, *MYB58*, and *MYB63*, are down-regulated (Zhang et al. 2018). CRY1 interacts with various proteins to regulate multiple developmental processes in plants and is located in both the nucleus and cytoplasm (Phee et al. 2007). In pears, four CRYs were identified, with PbCRY1a considered critical for mediating blue light-induced lignification in pear *calli* (Wang et al. 2022a). These findings highlight the significant role of blue light on lignification; however, the underlying transcriptional regulation network remains unknown. This study aims to explore the regulatory role of CRY-mediated blue light signaling in lignification and to identify the molecular hubs connecting stone cell development with blue light signals.

The lignin biosynthesis pathway has been extensively analyzed in model plants (Boerjan et al. 2003). Numerous transcription factors (TFs) involved in SCW thickening and lignification have been identified through gene editing and homeosis or heterologous transformation technologies (Taylor-Teeples et al. 2015; Zhong et al. 2008), with the NAC-MYB regulatory network recognized as fundamental to lignin biosynthesis (Ohtani and Demura 2019). In pears, based on the rapidly evolved high-throughput sequencing methodology over the past 15 years have led to the discovery of an increasing number of critical regulators in pear fruits (Zhang et al. 2023). The *NAC STONE CELL PROMOTING FACTOR* (*PbNSC*) has been identified as a critical positive NAC regulator of stone cell formation (Wang et al. 2021). However, the regulatory network of the stone cell complexes and their interactions remain fragmented.

To preliminarily understand the effect of different light quality on lignification, we previously treated pear *calli* with various lights and found that PbCRY1a-mediated blue light specifically induced cell wall lignification (Wang et al. 2022a). In this study, we also found that the stone cell formation was enhanced by supplementary with blue light. Notably, this study demonstrated that the PbCRY1a-mediated blue light signal regulates stone cell development via PbbHLH195 activation of the *PbNSC* in pear fruits. We confirmed that PbbHLH195 directly binds to the promoter of *PbNSC*, serving as a transcriptional hub between blue light signal and lignification, while also interacting with PbCRY1a. Our results provide novel insights into the molecular mechanism underlying lignin biosynthesis in response to blue light and provide valuable genetic targets for improving pear fruit quality.

## Results

### Blue light positively regulates stone cell formation in pear fruits

**Fig. 1:**
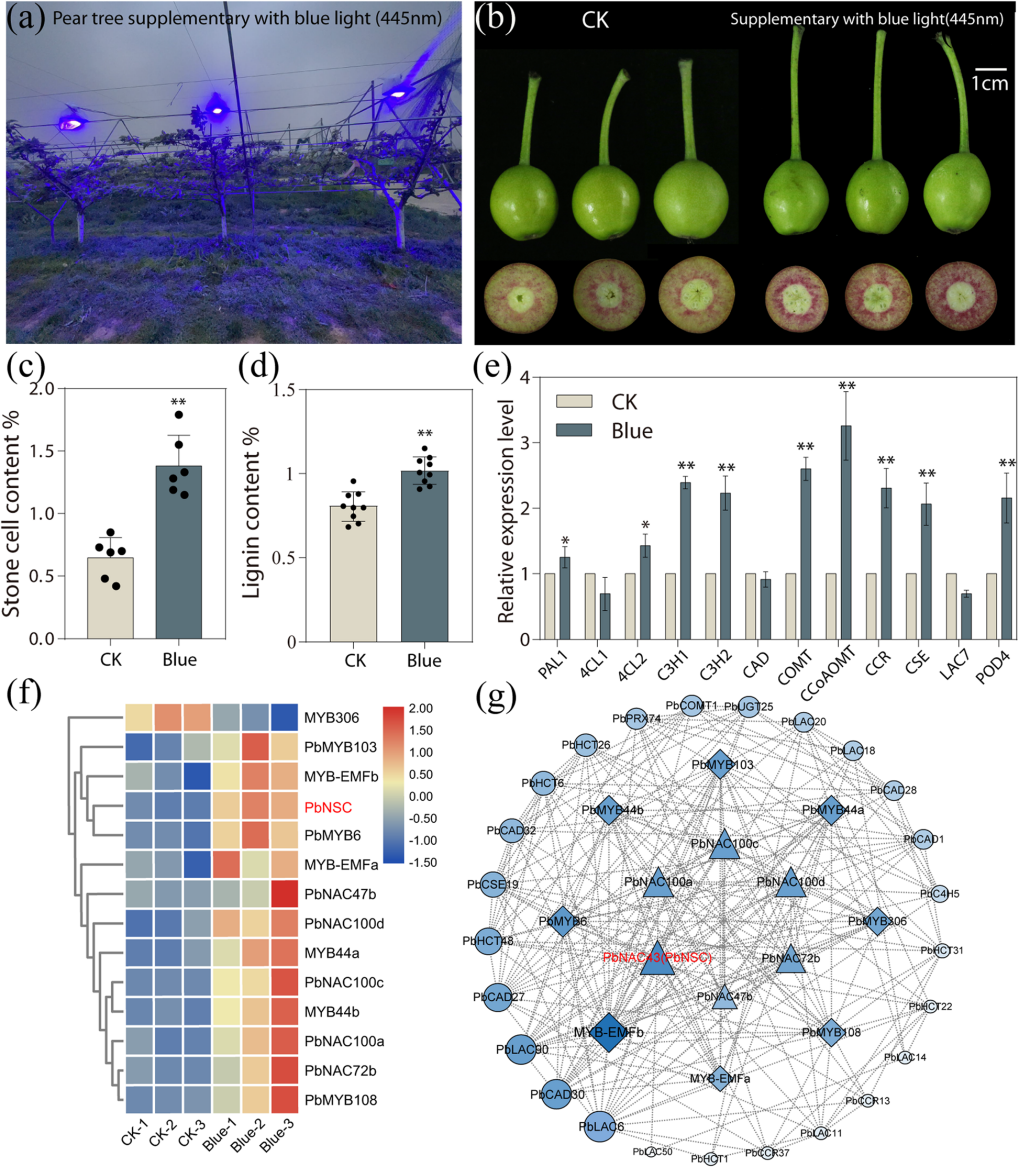
Blue light positively regulates stone cell formation in pear. **a** ‘Cuiguan’pear fruits supplemented with blue light. Phloroglucinol-HCL staining (**b**), stone cell content (**c**), and lignin content (**d**) in ‘Cuiguan’ pear fruits treated with blue light (445nm) at 35 DABF. (Bar = 5cm). (n ≥ 6). **e** Relative expression levels of TFs involved in stone cell formation and lignin biosynthetic genes in ‘Cuiguan’ pear fruits treated with blue light at 35 DABF (*n* = 3). **f** Heatmap of differentially expressed NACs and MYBs after blue light treatment based on RNA-sequencing data. **g** Co-expression network analysis illustrating correlations between MYB genes and lignin biosynthesis genes. Correlations are shown in degree, closeness, and betweenness algorithms

Blue light was supplemented to 'Cuiguan' (*Pyrus pyrifolia*) pear trees from 0 to 35 days after blossom fall (DABF), and fruit samples were collected at 35 DABF for subsequent analysis (Fig. 1a). Stone cells in the pear fruits were visualized using phloroglucinol-HCL staining (Fig. 1b). Pears grown under blue light conditions exhibited a deep red stain, indicating that supplementation with blue light significantly increased stone cell content. Further determination of stone cells and lignin content confirmed that blue light promoted the stone cells development (Fig. 1c, d). Moreover, the transcription levels of lignin biosynthetic genes were up-regulated (Fig. 1d). Additionally, lignin accumulation was found to be associated with carbohydrate and energy depletion through the shikimic acid pathway (Herrmann and Weaver 1999; Liu et al. 2019). Under blue light, sorbitol and glucose contents were significantly reduced, while the levels of three organic acid, including shikimic acid, a substrate of lignin biosynthesis, were increased (Fig. S1a, b). These physiological and biochemical changes caused by blue light influences pear stone cell formation.

Furthermore, RNA-sequencing analysis was performed to investigate the potential transcriptional regulation network underlying blue light-induced stone cell formation. A total of 630 differentially expressed genes (DEGs) were identified, including 447 upregulated and 183 downregulated genes (Fig. S2a).The phenylpropanoid biosynthesis pathway was significantly enriched (Fig. S2b, Fig. S3). Among these DEGs, 32 transcripts related to lignin biosynthesis were identified, including 30 upregulated and 2 downregulated transcripts (Table S1, Fig. S4). Additionally, the NAC-MYB cascades are widely considered to underpin in lignin biosynthesis (Ohtani and Demura 2019). In this study,we identified 6 NACs and 8 MYBs that were differentially expressed under blue light conditions (Fig. 1f). Co-expression network analysis revealed that *PbNSC*, identified as a critical ‘switch’ for lignin biosynthesis in stone cells (Wang et al. 2021), exhibited a strong correlation with lignin biosynthetic genes and differentially expressed MYBs under blue light irradiation (Fig. 1g). These findings suggest that blue light promotes lignification by modulating the transcription levels of downstream genes.

### Overexpression of *PbCRY1a* positively regulates stone cell formation

**Fig. 2:**
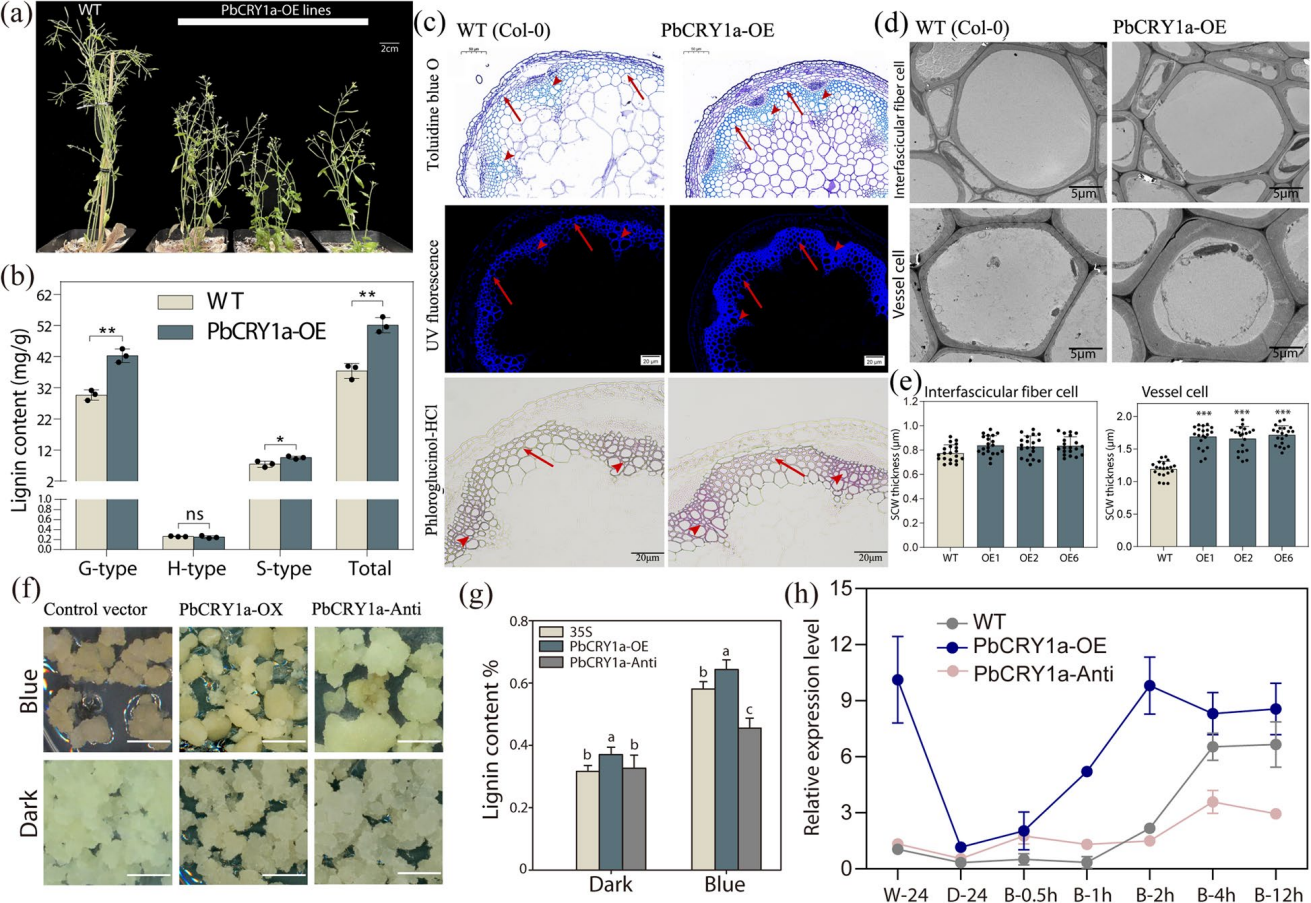
PbCRY1a-mediated blue light positively regulates stone cell formation in pear. **a** Phenotype of WT and PbCRY1a-OE Arabidopsis plants grown for 55 days under a long-day photoperiod. (Bar = 2 cm) (**b**) Lignin content in inflorescences stem and seeds of WT and PbCRY1a-OE plants. (*n* = 3). The ordinate represent the mean ± SD of the three biological replicates, with asterisks indicating significant differences as determined by a students-*t*-test (*p < 0.05, **p < 0.01). **c** Cross sections of WT and PbCRY1a-OE plants stained with Toluidine Blue O (Bar = 100 μm), Phloroglucinol-HCl (Bar = 50 μm), and UV-excited lignin autofluorescence (Bar = 20 μm). Arrows indicate fiber cells and arrowheads indicate vessel cells. **d** TEM analysis of interfascicular fiber and vessel cells in the stem of PbCRY1a-OE plants. Scare bar = 5 μm (**e**) Statistical analysis of SCW thickness in interfascicular fiber cells and vessel cells of WT and PbCRY1a-OE lines. Three plants in each line and more than 20 cells in each line were analyzed. OE, overexpression. **f** PbCRY1a-OE and antisense suppression (PbCRY1a-Anti) in pear *calli* treated with blue light and dark for 14 days (Bar = 1 cm) (**g**) Lignin content in PbCRY1a-OE and PbCRY1a-Anti transgenic *calli* treated with blue light and dark for 7 days. The error bars represent the means ± SD (*n* = 3). Vertical bars indicate the means ± SD of three biological replicates and lowercase letters indicate significant differences (two-tailed Student’s* t*-test (*p* < 0.01). **h** Induction of *PbNSC* expression by blue light. PbCRY1a-OE and PbCRY1a-Anti transgenic *calli* were transferred to darkness for 24 h, then treated with blue light to measure gene expression induction

Previous studies have shown that blue light-induced lignification is mediated by PbCRY1a (Wang et al. 2022a). In the study, overexpression of *PbCRY1a* (PbCRY1a-OE) in Arabidopsis resulted in a dwarf phenotype (Fig. 2a). qRT-PCR analysis confirmed the successful overexpression of PbCRY1a in the PbCRY1a-OE plants (Fig. S5a). The lignin content was significantly increased in transgenic lines, particularly G-lignin, which constitutes the primary component of lignin in stone cells (Jin et al. 2013) (Fig. 1b). Furthermore, paraffin sections stained with Toluidine Blue O, Phloroglucinol-HCl, and UV-excited lignin autofluorescence showed that the xylem tissue of the PbCRY1a-OE plants exhibited stronger staining and autofluorescence compared to the wild-type (WT) (Fig. 1c). Additionally, Transmission electron microscopy (TEM) analysis was performed to examine the anatomical features of the SCWs in PbCRY1a-OE plants (Fig. 1d). The results revealed that the SCWs of vessel cells in *PbCRY1a* transgenic plants was significantly thicker than those of Col-0 (Fig. 1e).

Furthermore, to investigate the cascading regulatory relationship between candidate regulators and light signaling, PbCRY1a-OE and antisense suppression (PbCRY1a-Anti) lines were employed in pear *calli* (Fig. 2f). qRT-PCR confirmed that PbCRY1a was successfully overexpressed in the PbCRY1a-OE lines and repressed in the PbCRY1a-Anti lines (Fig. S5b). Lignin content determination showed that downregulation of *PbCRY1a* significantly inhibited blue-light induced lignification, further confirming that blue-induced lignification is mediated by *PbCRY1a* (Fig. 2g). Subsequent qRT-PCR analysis revealed that blue light significantly induced the expression of the stone cell developmental switch *PbNSC*, while downregulation of *PbCRY1a* inhibited the blue light-induce upregulation *PbNSC* (Fig. 2h).

Overall, these findings reveal that PbCRY1a*-*mediated blue light signaling plays a crucial role in stone cell development by activating the developmental switch of stone cell formation.

### PbbHLH195 responds to blue light and directly binds to the promoter of *PbNSC*

**Fig. 3:**
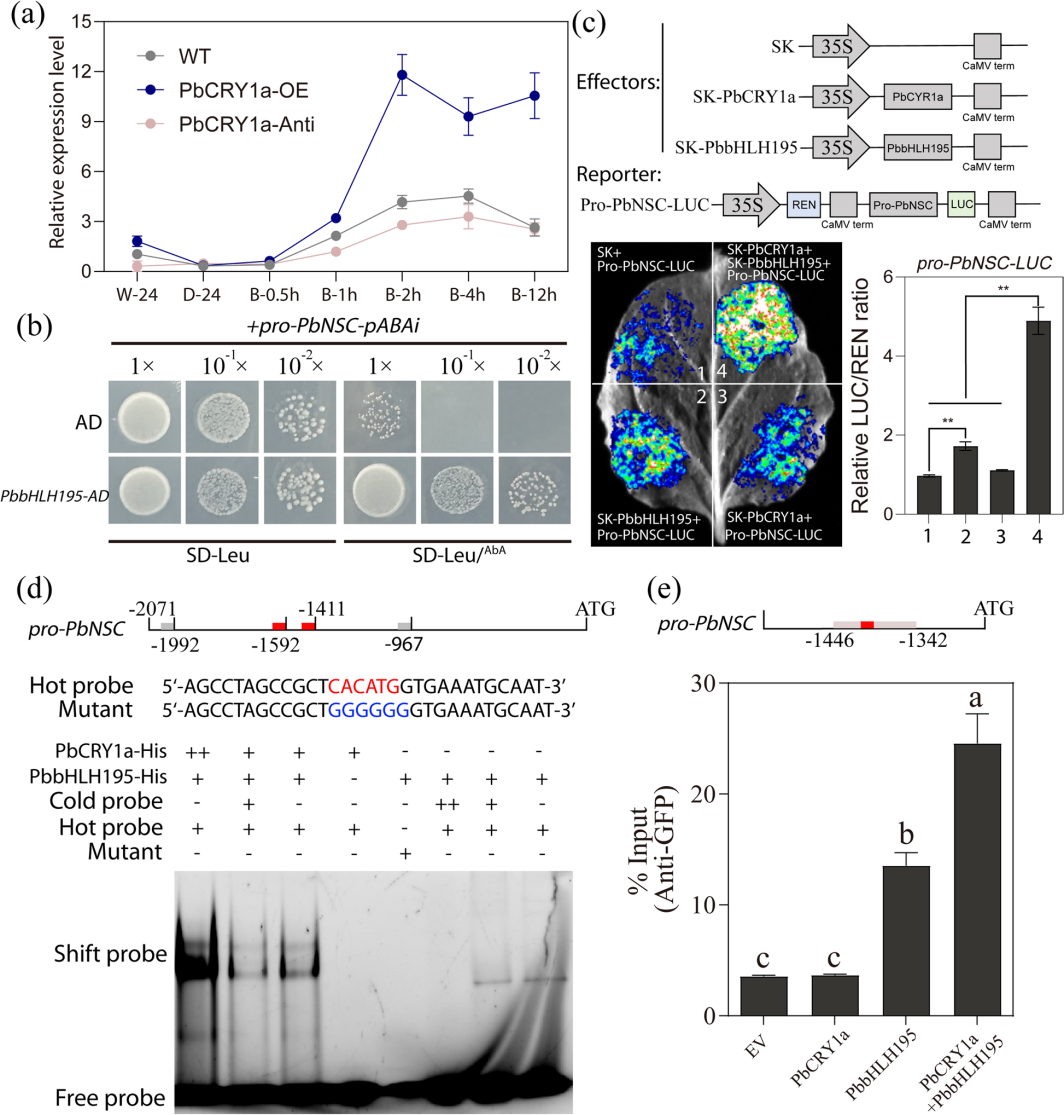
PbbHLH195 in response to blue light and directly binds to the *PbNSC* promoters. **a** Induction of *PbbHLH195* expression by blue light. WT and PbCRY1a-OE transgenic *calli* were transferred to darkness for 24 h, followed by blue light treatment to detect the induction of *PbbHLH195* expression. **b** Y1H assay showed that PbbHLH195 binds to the promoter of *PbNSC*. Specific binding analysis of PbbHLH195 to the *PbNSC* promoter in the Y1H system, with bait and either prey or negative control (bait/*pGADT7*) constructs co-transformed into yeast cells using SD/-Leu medium with or without AbA. **c** LUC assays demonstrating that PbCRY1a enhances the binding of PbbHLH195 to the *PbNSC* promoter. The ratio of LUC/REN from the empty vector (SK) plus the promoter was used as the reference (set as 1) for statistical analyses. The ordinate represents the mean ± SD of three biological replicates, and asterisks indicate significant difference as determined by students-*t*-test (**p < 0.01). **d** EMSA showed that PbCRY1a enhanced the binding of PbbHLH195 to the G-box sites in the *PbNSC* promoter. Bound and mutated nucleotides are indicated by red and blue letters, respectively. Purified His-tagged PbbHLH195 protein was incubated with unlabeled probe (cold) or biotin-labeled probe. The presence or absence of specific probes is marked by the symbol ‘ + ’ or ‘ − ’. Schematic structure of *PbNSC* with promoter fragments and cis-acting element, with G-box sites (− 1597 to − 1592, − 1416 to − 1411) highlighted in red and G-box-like sites (− 1997 to − 1992, − 972 to − 967) in gray. The primers used are listed in Table S4. **e** ChIP-qPCR analysis indicated that PbCRY1a enhanced the abundance of PbbHLH195 in the binding region of *PbNSC* promoter. The vertical bars represent the mean ± SD of three biological replicates with lowercase letters indicating significant differences determined by a two-tailed Student’s *t*-test (*p* < 0.01)

To elucidate the cascade switch of blue light-induced stone cell formation, the promoter of *PbNSC* was cloned and a yeast one-hybrid (Y1H) screening was used to identify potential transcriptional hubs linking stone cell development with blue light signal. Hundreds of positive clones were obtained from the pear flesh cDNA library, and subsequent sequencing and NCBI database blast analysis revealed eight TFs as potential upstream regulators of *PbNSC* (Table S3). Among these, a bHLH TF, denominated as *PbbHLH195*, was upregulated under blue light (Fig. S6). qRT-PCR analysis indicated that *PbbHLH195* was significantly induced by PbCRY1a-mediated blue light signaling (Fig. 3a). Thus, *PbbHLH195* is considered an upstream regulator of *PbNSC* in response to blue light.

To further verify the regulatory relationship between *PbbHLH195* and *PbNS*C, Y1H interaction assays were conducted. The results showed that PbbHLH195 directly induced the expression of the AbA resistance reporter gene driven by *PbNSC* promoter, indicating that PbbHLH195can bind directly to the *PbNSC* promoter (Fig. 3b). Dual luciferase (LUC) assays were conducted to verify the interactions. Tobacco leaves co-injected with *pro-PbNSC-LUC* and *pGreenII 62-SK* showed very low luminescence, while a significantly stronger luciferase signal was observed when co-injected with pro-PbNSC-LUC and SK-PbbHLH195. This fluorescence signal was further greatly enhanced by the addition of PbCRY1a protein (Fig. 3c). Additionally, bHLH TFs were reported to bind to either G-box or related motifs (Fernández-Calvo et al. 2011). Four putative binding sequences were identified in the *PbNSC* promoter. Subsequent Electrophoretic mobility shift assay (EMSA) analysis confirmed that PbbHLH195-His binds to the G-box (5’-CACATG-3’) elements in the *PbNSC* promoter (Fig. 3d Lane 1–4), Notably PbCRY1a couldn't bind to the promoter of *PbNSC*; instead, it enhanced the binding of PbbHLH195 to the *PbNSC* promoter (Fig. 3d, Lane 5–8). Furthermore, ChIP-qPCR analysis indicated that PbCRY1a increased the abundance of PbbHLH195 in the binding region of the *PbNSC* promoter. Overall, these findings reveal that PbCRY1a enhances the activation of PbbHLH195 on the *PbNSC* promoter*.* These results suggest that PbCRY1a-mediated blue light signaling regulates lignification in stone cells through the activation of *PbNSC* by PbbHLH195 in pear fruits.

### PbbHLH195 is essential for blue light-induced lignin biosynthesis, interacts with PbCRY1a

**Fig. 4:**
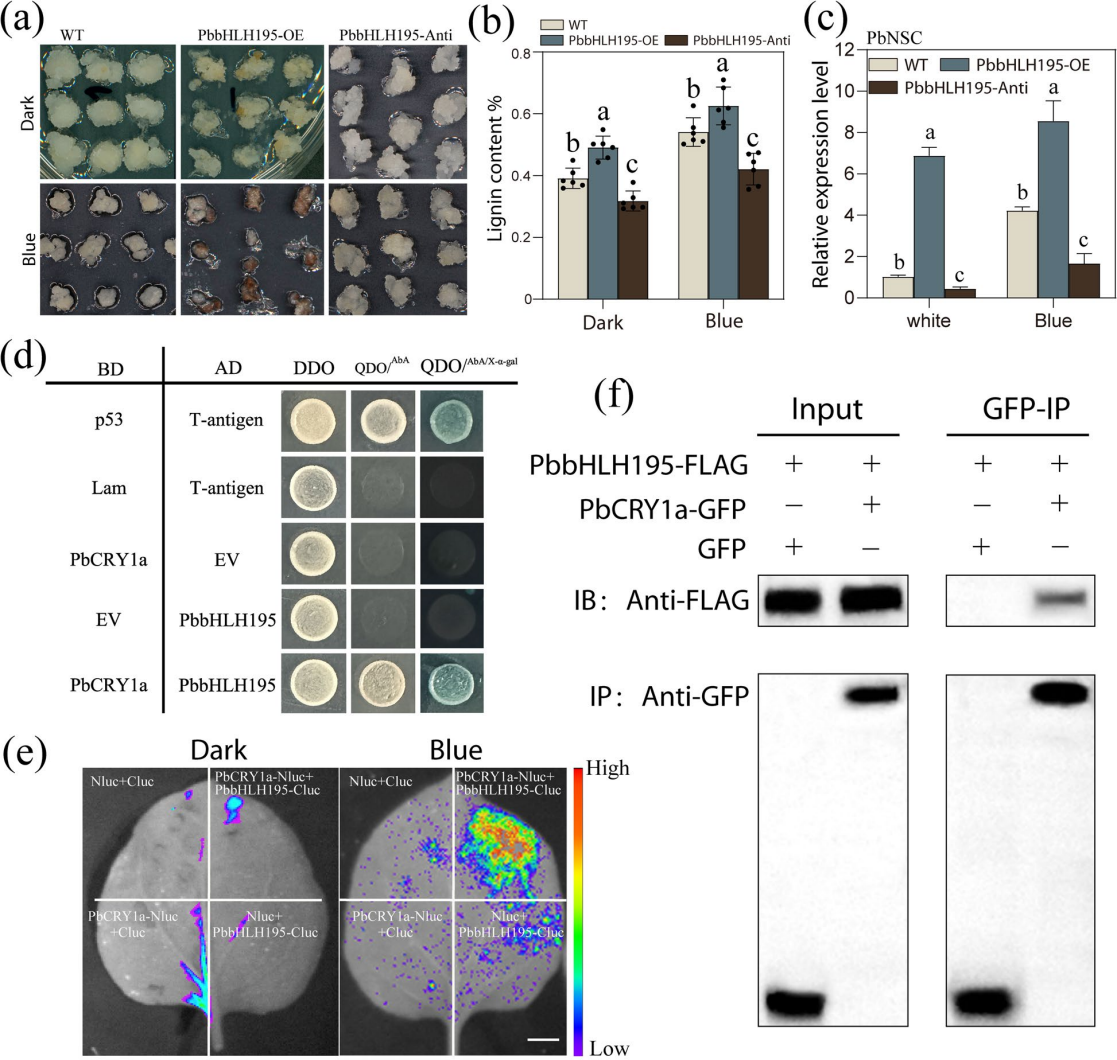
PbbHLH195 is essential for blue light-induced lignin biosynthesis and physically interacts with PbCRY1a. **a** Overexpression of *PbbHLH195* (PbbHLH195-OE) and antisense suppression (PbbHLH195-Anti) in pear *calli* treated with blue light and dark for 14 days (Bar = 1 cm). **b** Lignin content in PbbHLH195-OE and PbbHLH195-Anti transgenic *calli* treated with blue light and dark for 7 days (*n* = 3). **c** Relative expression analysis of *PbNSC* after overexpression and suppression of *PbbHLH195*. (*n* = 3). The vertical bars represent the mean ± SD of three biological replicates and lowercase letters indicate significant differences by two-tailed Student’s* t*-test (*p* < 0.05). **d** Yeast two-hybrid (Y2H) assay showing the interaction between PbCRY1a and PbbHLH195. The transformed yeast cells were grown on DDO (SD-Trp/-His) and QDO (SD-Trp/-Leu/-His/-Ade/) medium. p53 (*pGBKT7-53*) + AD-T (*pGADT7-T*) were used as a positive control; BD-Lam (*pGBKT7-Lam*) + AD-T (*pGADT7-T*) were used as a negative control. EV, empty vector. **e** Split-luciferase complementation imaging (LCI) assays showing the interactions between PbCRY1a and *PbbHLH195* in tobacco leaves. The interaction was observed in *N. benthamiana* leaves under darkness and blue light (200 μmol/m^2^/s). Bars = 1 cm. Empty vectors were used as negative controls. **f** co-immunoprecipitation (CoIP) assay showing the interaction between PbCRY1a and PbbHLH195. PbCRY1a-GFP and PbbHLH195-FLAG were co-transformed into *N. benthamiana* protoplasts. After transformation, the *N. benthamiana* plants were growth under continuous blue light. Proteins were extracted from protoplasts, immunoprecipitated using anti-FLAG matrix beads, and analyzed by western blotting using anti-GFP or anti-FLAG antibodies

The results described above highlight the importance of the ‘PbbHLH195-PbNSC’ cascade in response to blue light in regulating stone cell formation. To further analyze the role of *PbbHLH195* between stone cell development and blue light signal, the overexpression of *PbbHLH195* (PbbHLH195-OE) and antisense suppression (PbbHLH195-Anti) lines were generated in pear *calli* (Fig. 4a). qRT-PCR confirmed that PbCRY1a was successfully overexpressed in the PbCRY1a-OE lines and repressed in the PbCRY1a-Anti lines (Fig. S7). We found that the suppression of *PbbHLH195* transcription significantly inhibited blue-induced lignification in transgenic pear *calli* (Fig. 4b), indicating the essential role of *PbbHLH195* in blue light signaling. Additionally, the expression of *PbNSC* was upregulated in PbbHLH195-OE *calli*, while the suppression of *PbbHLH195* inhibited the induction of *PbNSC* by blue light (Fig. 4c). These results indicate that PbbHLH195 is essential for blue light-induced lignification.

To better understand the interaction between PbbHLH195 and blue light signal, subsequent yeast two-hybrid (Y2H) assay revealed that PbCRY1a interacts directly with PbbHLH195 (Fig. 4d). Further The split-luciferase complementation imaging (LCI) assays showed strong interaction signals following co-expression of PbCRY1a and PbbHLH195 under continuous blue light, while no signal was detected in the negative controls and *N. benthamiana* leaves under darkness (Fig. 4e). These results indicate that PbCRY1a interacts with the PbbHLH195 protein in a blue light-dependent manner. To further confirm this interaction, the co-immunoprecipitation (CoIP) assay was conducted, revealing that the PbbHLH195-FLAG fusion protein was precipitated by PbCRY1a-GFP in *N. benthamiana* plants grown under continuous blue light condition (Fig. 4f). Based on these results, we conclude that *PbbHLH195* acts as a novel transcriptional hub connecting the blue light signal with lignification in pear stone cells.

### Overexpression of *PbbHLH195* positively regulates SCW thickening and lignin deposition

**Fig. 5:**
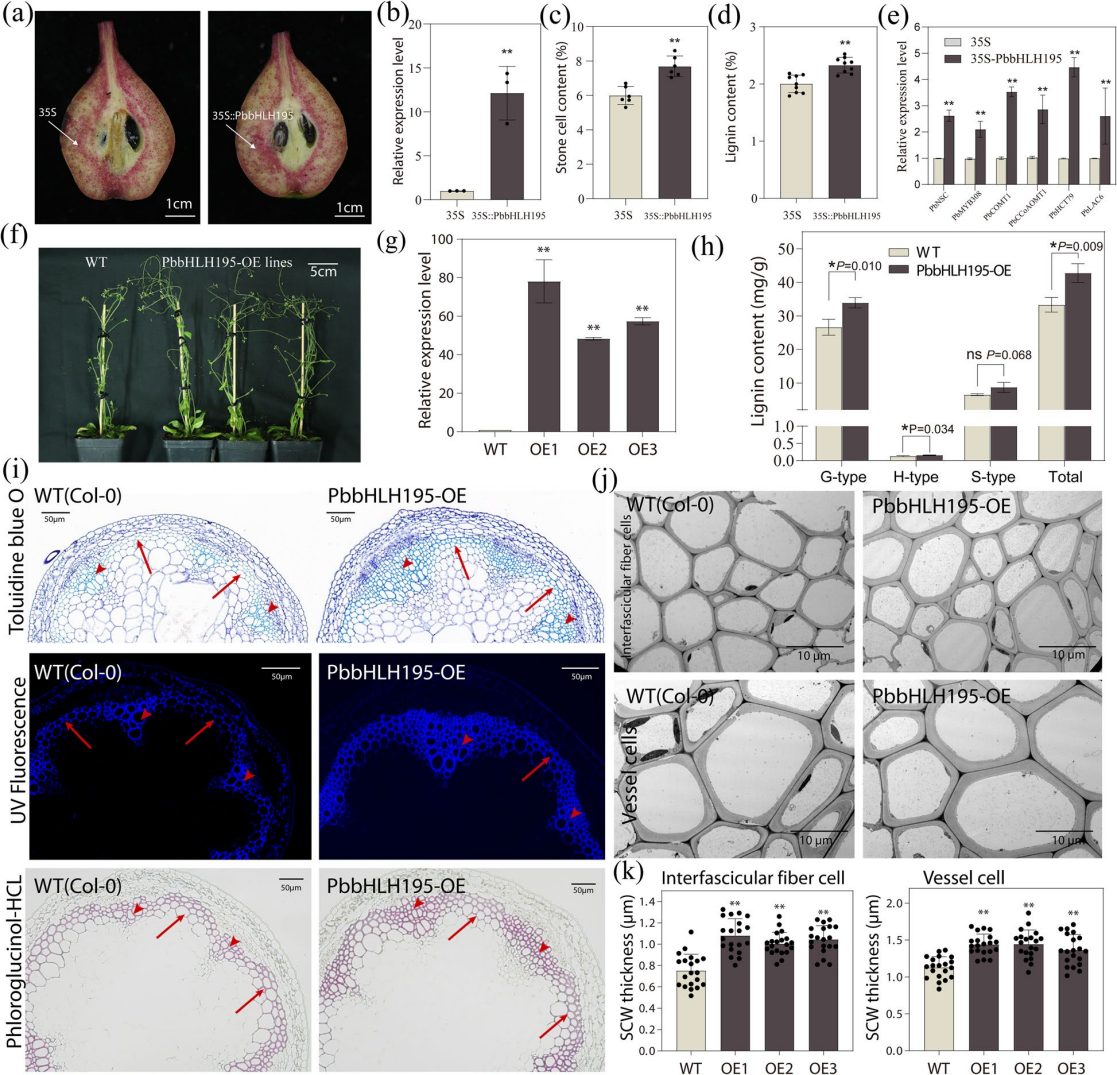
Functional validation of *PbbHLH195*. **a** Phloroglucinol-HCL staining of ‘Dangshansuli’ pear fruits after 7 days of *PbbHLH195* overexpression. *35S* represent the empty vector control, and *35S-PbbHLH195* represents overexpression of *PbbHLH195* mediated by the *35S* strong promoter. Scale bars = 1 cm. **b** Relative expression of *PbbHLH195* 7 days after injection. (*n* = 3). **c**-**d** Stone cell (**c**) and lignin (**d**) content of pear fruits 7 days after injection (*n* = 6). **e** Expression analysis of lignin biosynthetic genes in pear fruits 7 days after injection. Error bars represent the mean ± SD (*n* = 3). **f** Phenotype of Col-0 and PbbHLH195-OE Arabidopsis plants grown for 55 days under a long-day photoperiod. Scale bars = 5cm (**g**) qRT-PCR analysis showed the overexpression of *PbbHLH195* in representative overexpression lines (*n* = 3). **h** Lignin content in inflorescences stems of WT and PbbHLH195-OE Arabidopsis plants. (*n* = 3). **i** Cross sections of WT and PbbHLH195-OE Arabidopsis plants stained with Toluidine Blue O, Phloroglucinol-HCl, and UV-excited lignin autofluorescence. Arrows indicate fiber cells; arrowheads indicate vessel cells. Scale bars = 50 μm. **j** TEM of cross-sections of interfascicular fiber and vessel cells in 55-day-old inflorescence stems. Scare bar = 10 μm. **k** Statistical analysis of SCW thickness in interfascicular fiber cells and vessel cells of Col-0 and *PbbHLH195* transgenic lines. Three plants per line and more than 20 cells per plant were analyzed. OE, overexpression. Error bars represent the means ± SD of the three biological replicates, and asterisks indicate the significant difference of the students-*t*-test (*p < 0.05, **p < 0.01)

To further understand the biological function of *PbbHLH195* in stone cell formation, transient overexpression was performed in ‘Dangshansuli’ (*Pyrus bretschneideri* Rehd.) fruitlets. Increased in stone cell staining was detected at the infiltration sites in the *PbbHLH195* overexpression samples (Fig. 5a). qRT-PCR confirmed that *PbbHLH195* was successfully overexpressed at the *35S-PbbHLH195* injection site (Fig. 5b). Stone cells and lignin content were significantly increased after overexpression of *PbbHLH195* (Fig. 5c, d). In addition, overexpression of *PbbHLH195* increased the expression levels of *PbNSC* and lignin biosynthesis genes, including *PbCOMT1* and *PbLAC6* (Fig. 5e).

Next*, PbbHLH195* overexpressing Col-0 Arabidopsis lines (PbbHLH195-OE) were generated by transformation with a *p35S-PbbHLH195* fusion construct. The homozygous plants from the T3 generation were used for functional validation (Fig. 5f). qRT-PCR confirmed that *PbbHLH195* was successfully overexpressed in the transgenic lines (Fig. 5g). Subsequent lignin content determination showed a noticeable increase in the G- and H-lignin content of the inflorescence stems (Fig. 5h). Furthermore, cross sections of WT and PbbHLH195-OE plants stained with Toluidine Blue O, Phloroglucinol-HCl, and UV-excited lignin autofluorescence indicating that the lignified tissue of the transgenic lines exhibited stronger staining than that of the WT. Both the vessels cell and interfascicular fiber cell of PbbHLH195-OE exhibited stronger auto-fluorescence signals of lignin compared to WT plants (Fig. 5i). Additionally, TEM analysis was conducted to detect the anatomical features of the SCWs in PbbHLH195-OE plants (Fig. 5j). The results indicated that the SCWs of interfascicular fiber cells and vessel cells of *PbbHLH195* transgenic plants was significantly thicker than those in WT plants (Fig. 5k). Furthermore, we found that the expression of *AtSND1* (the homologous gene of *PbNSC* in Arabidopsis) in *PbbHLH195* transgenic plants was significantly induced by overexpression *PbbHLH195* (Fig. S8), indicating that the PbbHLH195- PbNSC module was conserved between pear and Arabidopsis. Overall, these findings reveal that *PbbHLH195* promotes lignification and SCW thickening during stone cell development.

## Discussion

The development of lignin was known as an essential event during vascular plants evolved from aquatic to terrestrial environments. Lignin provides rigidity and hydrophobicity to SCWs, enabling plants to transport water and nutrients over long distances and grow tall (Somerville et al. 2010). Lignin also provides structural support, protects plant cells from environmental stress, and acts as a barrier (Zhao et al. 2023). The NAC-MYB regulatory network has been identified as a play role in lignin biosynthesis (Ohtani and Demura 2019). Stone cells in pear fruit, which consist entirely of lignified SCWs, negatively affect fruit quality. Environmental signals influences lignification by activating receptors or transcriptional hubs that first-layer developmental switches (Ohtani and Demura 2019). Our findings not only contribute to the understanding of novel molecular hubs that connect environmental and developmental signals, but also provide new insight for the improving of pear fruit quality.

LEDs are increasingly used to optimize crop quality and production, by adapting light spectrum to plant needs through precise control of the spectral composition of supplemental light. For example, UV LEDs are often used to improve plant defense, as natural UV light does not penetrate greenhouses (Prieto-Ruiz et al. 2019). Blue light triggers specific biochemical and physiological processes, leading to a higher accumulation of epidermal flavanols in pepper (Hoffmann et al. 2015). In this study, we found that blue light specially affects the lignification of stone cells. In the future, specialized fruit bags or filters could be developed to regulate fruit quality by modifying the light spectrum, based on our findings. However, further field trials are necessary to validate this approach. The first-layer NAC genes involved in SCW thickening and lignification are regulated by phytohormonal or environmental signals and function as molecular switches for lignin biosynthesis (Moura et al. 2010). For example, gibberellins (GA) regulate SCW biosynthesisby relieving the interaction between the DELLA protein SLR1 and the top-layer TFs NAC29/31, which are critical for SCW formation (Huang et al. 2015). In Arabidopsis, blue light promotes SCW thickening by activating the direct binding of bHLH TF family gene MYC2/MYC4 to NST1 (Zhang et al. 2018), while far-red light inhibits SCW thickening through the PIF4-MYC2/MYC4 module (Luo et al. 2022). In this study, blue light supplementation increased the expression of *PbNSC*, the lignification switch in stone cell formation. Therefore, identifying the regulatory factors acting upstream of PbNSC was a critical objective of this study. A set of putative regulators were identified through Y1H screening, including *PbbHLH195* (*Pbr042466.1*), *PbERF105/014* (*Pbr001362.1/Pbr032206.1*), *PbHD19* (*Pbr019133.1*), *PbbZIP43* (*Pbr022222.1*), and *PbPIF4* (*Pbr029607.1*) (Table S3). These TFs may play a role in lignin biosynthesis in stone cells through other environmental or hormonal signals. Elucidating these hubs will help us control lignification, harmonize it with plant development and environmental adaptation.

In this study, we identified *PbbHLH195* as a novel molecular hub connecting blue light signaling with stone cell developmental switches. Cryptochrome-mediated blue light regulates cell lignification via the activation of the PbNSC module by *PbbHLH19*5 in pear fruits. The bHLH gene family is one of the largest TF families in plants, which has pleiotropic regulatory roles in plant growth and development, environmental and stress response, and plant hormone signaling (Gao and Dubos 2023). Many bHLH TFs have been reported to participate in light signaling by inducing the expression of downstream regulators. For example, the CRYPTOCHROME-INTERACTING BASCI-HELIX-LOOP-HELIX protein (CIB1) interacts with CRY2 activate FT transcription (Liu et al. 2008). The bHLH TF *MYC2* isessential for blue light responses in Arabidopsis seedling development (Srivastava et al. 2022). Furthermore, *BRASSINOSTEROID ENHANCED EXPRESSION 1* (*AtbHLH44*) physically interacts with *AtCRY2* to promote flowering in a blue light dependent manner (Wang et al. 2019). AtbHLH136/164/163 are direct targets of AtPIF4 and are involved in regulation the light, GA, and BR signaling pathways (Bai et al. 2012). Additionally,* AtbHLH165/166* have been illustrated as a connectors between light and auxin responsiveness (Roig-Villanova et al. 2007).

In addition, increasing evidence suggests that bHLH family members are involved in lignification. In chrysanthemum, the bHLH protein CmHLB regulates lignification by interacting with CmKNAT7 (Zhao et al. 2022). The ‘EjbHLH14-EjHB1-EjPRX12’ cascade has been shown to participate in chilling-induced lignin deposition in loquat fruit (Zhang et al. 2022). In rice, overexpression *OsbHLH034* increased lignin accumulation in response to jasmonic acid (JA) (Onohata and Gomi 2020). In this study, *PbbHLH195* was identified as a novel molecular regulator of lignification and SCW thickening via the activation of PbNSC. The evidence presented here shows that lignin accumulation in stone cells is largely regulated by PbCRY1a-mediated blue light signal through PbbHLH195-PbNSC module. These findings not only advance our knowledge of blue light-induced lignification, but also bridge the gap in our understanding of how photocontrol can enhance fruit quality. Understanding the underlying mechanisms is crucial for optimizing the performance of horticultural plants, ultimately contributing to the development of resilient crop-production systems.

## Materials and methods

### Plant materials

The ‘Cuiguan’ pear trees used for blue light treatment were grown in an experimental teaching base located in Baima town, Lishui District of Nanjing, China. Pear trees under uniform cultivation without any visible disease or insect infection were selected randomly. These selected pear trees were and exposed to blue light using 445 nm LED lights produced by Aijia Electronic Technology (Xuzhou, Jiangsu, China). The blue light irradiation is supplied for ten hours every day, starting from 0 until 35 DABF. After harvesting, the collected samples were immediately cut and frozen in liquid nitrogen, and then stored at -80℃. ‘Dangshansuli’ used for transient overexpression were harvested from a garden on Hushu Street, Jiangning District, Nanjing at 35 DABF. Additionally, WT *Arabidopsis thaliana* accession Col-0 preserved in the laboratory, was used for heterologous transformation analyses.

### Determination of physiological and biochemical data

The contents of stone cell and acetyl bromide lignin were measured using the method described by (Tao et al. 2009). For the determination of lignin monomers in transgenic Arabidopsis was conducted using a Trace1310 ISQ Gas chromatograph Mass spectrometer (GC–MS) (Thermo, Wilmington, USA), following the protocol outlined by (Lapierre et al. 1995).

The extraction of soluble sugars and organic acid components from pear fruits to the method described by (Wu et al. 2022). The soluble sugar components, including fructose, sorbitol, glucose, and sucrose, were analyzed using an ACQUITY H-Class UPLC system (Waters, USA). Meanwhile the organic acid components were detected using an UltiMate 3000 UPLC system (Thermo, USA).

### Co-expression network and qRT-PCR analysis

The FPKM (Fragments Per Kilobase Million) values generated by RNA-sequencing was used to explore the expression patterns of differential expressed genes. The measurement of expression similarity between gene pairs was characterized by Pearson’s correlation coefficient values. These values were then filtered using Excel software. Correlations were analyzed using degree, closeness, and betweenness algorithms (Han et al. 2021). Data visualization was performed with Cytoscape software (Shannon et al. 2003). qRT-PCR analysis was performed based on the method described previously (Wang et al. 2022a). The 2^−ΔΔCT^ method was used to calculate the relative expression levels (Livak and Schmittgen 2001).

### Transient transformation of pear fruits

The *p35S-PbbHLH195-GFP* fusion construct was obtained by inserting the fragment into the *pCAMBIA1300-35S-GFP* vector. The plasmids were transferred to the *A. tumefaciens* strain GV3101. The infection solution (200 mM MES and, MgCl_2_, 200 μM Acetosyringone, OD_600_ = 0.8, pH = 5.6) was used to resuspended *Agrobacterium* cells. Transient transformation were conducted based on the method described previously (Wang et al. 2022a). After 7 days, samples were collected. The infiltration sites were harvested and immediately frozen in liquid nitrogen before being preserved at -80°C.

### Arabidopsis transformation

Agrobacterium cells containing *p35S-PbCRY1a-GFP* and *p35S-PbbHLH195-GFP* fusion constructs were resuspended in the infection solution (10% sucrose, 200 μM Acetosyringone, 0.005% surfactant Silwet L-77, OD_600_ = 0.8, pH = 5.7) (Li et al. 2023). The floral-dip method mediated by Agrobacterium was used to obtain the overexpression lines of PbCRY1a and PbbHLH195 using Arabidopsis Col-0 (Clough and Bent 1998). Homozygous plants were obtained through three generations of antibiotic screening using 25 mg/L hygromycin B.

### Pear calli transformation

The coding sequences of *PbCRY1a* and *PbbHLH195* were cloned into the *pCAMBIA1300-GFP* vector to generate *35S-PbCRY1a* and *35S-PbbHLH195* overexpression vectors. The specific fragments of *PbCRY1a* and *PbbHLH195* were inserted in reverse into the *pCAMBIA1300-GFP* vector to create the 35*S-PbCRY1a-Anti* and *35S-PbbHLH195-Anti* antisense suppression vectors. The transformation of pear *calli* was performed using *Agrobacterium*-mediated transformation, following the methods described by (Wang et al. 2022a).

### Paraffin cross section

Samples of the pear flesh and Arabidopsis inflorescence stems were removed using a sharp blade and fixed in FAA solution for more than 24 h. Gradient alcohol solutions (50%, 70%, 80%, 95%) was used for dehydratation. The pear flesh tissue was then embedded in paraffin wax and cooled to -20 ◦C. Stone cell staining was performed using a Safranine O-Fast Green staining solution, while the Arabidopsis inflorescence stems was stained with Toluidine Blue O and Phloroglucinol-HCl. Finally, the tissue sections were mounted by neutral balsam, and a microscope obtained the images.

### TEM

Samples were excised from Arabidopsis stem using a sharp blade and immediately placed in fixation fluid for 1 to 2 h (4 °C). The samples were then fixed in 2% osmic acid solution and dehydrated using gradient alcohol. They were embedded in Spurr’s epoxy resin for 6 h and stained with 1% KMnO_4_. TEM observation was performed with a Tecnai F20 at 220 kV.

### Y1H assays

The promoter sequences were inserted into the *pAbAi* empty vector to generate the *pro-PbNSC-pAbAi* fusion construct. The fusion construct was then transformed into theY1HGold yeast strain. The coding sequences of *PbbHLH195* were inserted into the *pGADT7* (AD) prey vector, resulting in the generation of the *AD-PbbHLH195* fusion construct, which was transformed into individual bait-reporter yeast strains. Interaction verification was performed according to the method described by (Wang et al. 2022b; Ren et al. 2023).

### LUC assays

The coding sequence of the *PbbHLH195* was inserted into the *pGreenII-62SK* vector (SK), and the promoter sequence of *PbNSC* was cloned into the *pGreenII 0800-LUC* vector. The plasmids were extracted from the fusion constructs and the empty vectors. All of the fusion constructs were transformed into the GV3101-pSoup *Agrobacterium* strain. LUC assays were conducted on tobacco leaves by infiltration cell suspensions mixtures using a needle-free syringe, according to the previously described (Wang et al. 2022b).

### EMSA

The coding sequence of *PbbHLH195* was cloned from plasmids and inserted into the *pCOLD-TF* vector to generate the fusion construct. HIS Sefinose resin (Sangon Biotech, China) was used to purify the fusion proteins. The binding reaction was carried out in a 0.2 mL centrifugal tube at room temperature for 20 min. One microliter of the fluorescently labeled probe and the fusion proteins were incubated in a 10μL reaction solution (1 mg poly (dI·dC), 10 mM MgCl_2_, 60 mM KCl, and 0.05% NP-40) at 25 °C for 20 min. For probe competition, competing non-labeled probes were added to the reaction mixture at relative concentrations of 10X and 20X. Subsequently, the DNA–protein complexes were separated on a non-denaturing polyacrylamide gel using the pre-cooled TBE buffer. Finally, the ChemiDoc XRS + system was used for scanning (BioRad, Hercules, USA).

### ChIP-qPCR

The ChIP assay was performed as previously described (Alabd et al. al., 2022). The pear *calli* (5 g) were collected from 20-day-old transgenic calli containing GFP alone, *PbCRY1a-GFP*, and *PbbHLH195-GFP* and then incubated in freshly prepared 1% (w/v) formaldehyde for 15 min at room temperature under vacuum conditions. The chromatin DNA was extracted and fragmented to approximately 104bp using a sonication process with the Bioruptor Plus device (Diagenode) at 4 °C for 30 min (30 s on, followed by 30 s off). The sonicated chromatin was immunoprecipitated overnight using an anti-GFP antibody (Abcam, China). To reverse the cross-linking, the solution was incubated at 65 °C overnight, after which the DNA was extracted and analyzed by qPCR. Details regarding the primer sequences are provided in Supplementary Table S4.

### Protein interactions

For the Y2H assay, the coding sequences of *PbCRY1a* and *PbbHLH195* were inserted into the *pGBKT7* (BD) and *pGADT7* (AD) vectors, respectively, via the restriction sites *Eco*R I and *Bam*H I. The resulting fusion constructs were then transformed into the Y2HGold yeast strain. The transformed Y2HGold cells were cultivated on SD/-Trp/-Leu/-His/-Ade/ + AbA + X-α-gal medium 300 ng/mL AbA and 30 μg/mL X-α-gal at 28◦C for 2–3 days to test the interactions.

The LCI assays were conducted on *Nicotiana benthamiana* leaves by infiltration of cell suspensions mixtures using a needle-free syringe. The transformed tobacco plants were cultured in the dark and blue light (200 μmol/m^2^/s) for 3 days. Images were captured using a CCD camera (PIXIS: 1024B) and IndiGO software.

CoIP assay was performed by co-transformed into *N. benthamiana* leaves. Total protein extraction was conducted 3 days after injection using 600 μL of plant IP buffer containing a protease inhibitor cocktail (Roche). Then, 500 μL of the extract was incubated with anti-FLAG beads (SIGMA) for 4 h at 4℃. The eluate was subjected to western blotting using anti-GFP and anti-FLAG antibodies according to the illustration described previously (Lin and Lai 2017; Li et al. 2024).

### Data analysis

All data are presented as the mean values of at least three biological replicates and visualized using SigmaPlot 10.0 and Graphpad prism 9.0 software. Statistical analysis, including Student’s *t*-tests, were conducted using SPSS 18.0 software (SPSS Inc., city, USA). The error bars represent the means ± SD of at least three biologicals replicates, with asterisks indicating significant differences analyzed by two-tailed Student’s *t*-test (*p < 0.05, **p < 0.01). The cluster heatmaps were performed by TBtools software (Chen et al. 2020).

## Supplementary Information

Supplementary Material 1.

Supplementary Material 2. Figure S1. Physiological changes of pear fruit after blue light treatment. Contents of plant hormones (a), soluble sugars (b), and organic acids (c) of ‘Cuiguan’ fruits under blue light irradiation (*p < 0.05, **p < 0.01). Figure S2. Statistical and enrichment analysis of differentially expressed genes. (a) Volcano plot of differentially expressed genes. (b) KEGG enrichment of differentially expressed genes. Figure S3. KEGG classification analysis of differentially expressed genes. Figure S4. Heatmap cluster analysis of lignin biosyhthetic genes in pear fruits after blue light treatment. Figure S5. qRT-PCR verification of gene overexpression and repression (a) qRT-PCR analysis showed the overexpression of *PbCRY1a *in representative overexpression Arabidopsis lines. (b) qRT-PCR confirmed that *PbCRY1a* was successfully overexpressed at the PbCRY1a-OE lines, and repressed at PbCRY1a-Anti lines. The ordinate was the mean ± SD of the three biological replicates, and asterisk indicates the significance difference of the students-*t*-test (**p < 0.01). Figure S6. Heatmap cluster indicates the differentially expressed TFs of pear fruits under blue light based of RNA-sequencing data. Figure S7. qRT-PCR confirmed that *PbbHLH195* was successfully overexpressed at the PbbHLH195-OE lines, and repressed at PbbHLH195-Anti lines. The ordinate was the mean ± SD of the three biological replicates, and asterisk indicates the significance difference of the students-*t*-test (**p < 0.01). Figure S8. The relative expression of *AtSND1 *(homology of *PbNSC*) was significantly induced by overexpression *PbbHLH195*. The ordinate was the mean ± SD of the three biological replicates, and asterisks indicates the significance difference of the students-*t*-test (**p < 0.01).

## Data Availability

The raw transcriptome datasets are available in the National Center for Biotechnology Information (NCBI, https://www.ncbi.nlm.nih.gov/) under the BioProject number PRJNA1062740.

## Abbreviations

SCW: Secondary cell wall
qRT-PCR: Quantitative real-time polymerase chain reaction
TFs: Transcription factors
Y1H: Yeast one-hybrid assay
Y2H: Yeast two-hybrid assay
EMSA: Electrophoretic mobility shift assay
TEM: Transmission electron microscopy
LUC: Dual luciferase
LCI: Split-luciferase complementation imaging
CoIP: Co-immunoprecipitation
OE: Overexpression
