# Supplementary material for: Cryptochrome-mediated blue light regulates cell lignification via PbbHLH195 activation of the PbNSC in pear fruits
Source: Mol Hortic. 2025 May 7;5:27. doi: 10.1186/s43897-025-00149-z (PMC12057157; doi:10.1186/s43897-025-00149-z)
Supplement: Supplementary file 2 — Supplementary Material 2. Figure S1. Physiological changes of pear fruit after blue light treatment. Contents of plant hormones (a), soluble sugars (b), and organic acids (c) of ‘Cuiguan’ fruits under blue light irradiation (*p < 0.05, **p < 0.01). Figure S2. Statistical and enrichment analysis of differentially expressed genes. (a) Volcano plot of differentially expressed genes. (b) KEGG enrichment of differentially expressed genes. Figure S3. KEGG classification analysis of differentially expressed genes. Figure S4. Heatmap cluster analysis of lignin biosyhthetic genes in pear fruits after blue light treatment. Figure S5. qRT-PCR verification of gene overexpression and repression (a) qRT-PCR analysis showed the overexpression of PbCRY1a in representative overexpression Arabidopsis lines. (b) qRT-PCR confirmed that PbCRY1a was successfully overexpressed at the PbCRY1a-OE lines, and repressed at PbCRY1a-Anti lines. The ordinate was the mean ± SD of the three biological replicates, and asterisk indicates the significance difference of the students-t-test (**p < 0.01). Figure S6. Heatmap cluster indicates the differentially expressed TFs of pear fruits under blue light based of RNA-sequencing data. Figure S7. qRT-PCR confirmed that PbbHLH195 was successfully overexpressed at the PbbHLH195-OE lines, and repressed at PbbHLH195-Anti lines. The ordinate was the mean ± SD of the three biological replicates, and asterisk indicates the significance difference of the students-t-test (**p < 0.01). Figure S8. The relative expression of AtSND1 (homology of PbNSC) was significantly induced by overexpression PbbHLH195. The ordinate was the mean ± SD of the three biological replicates, and asterisks indicates the significance difference of the students-t-test (**p < 0.01). [file 43897_2025_149_MOESM2_ESM.doc]

**Supplementary figures**

**
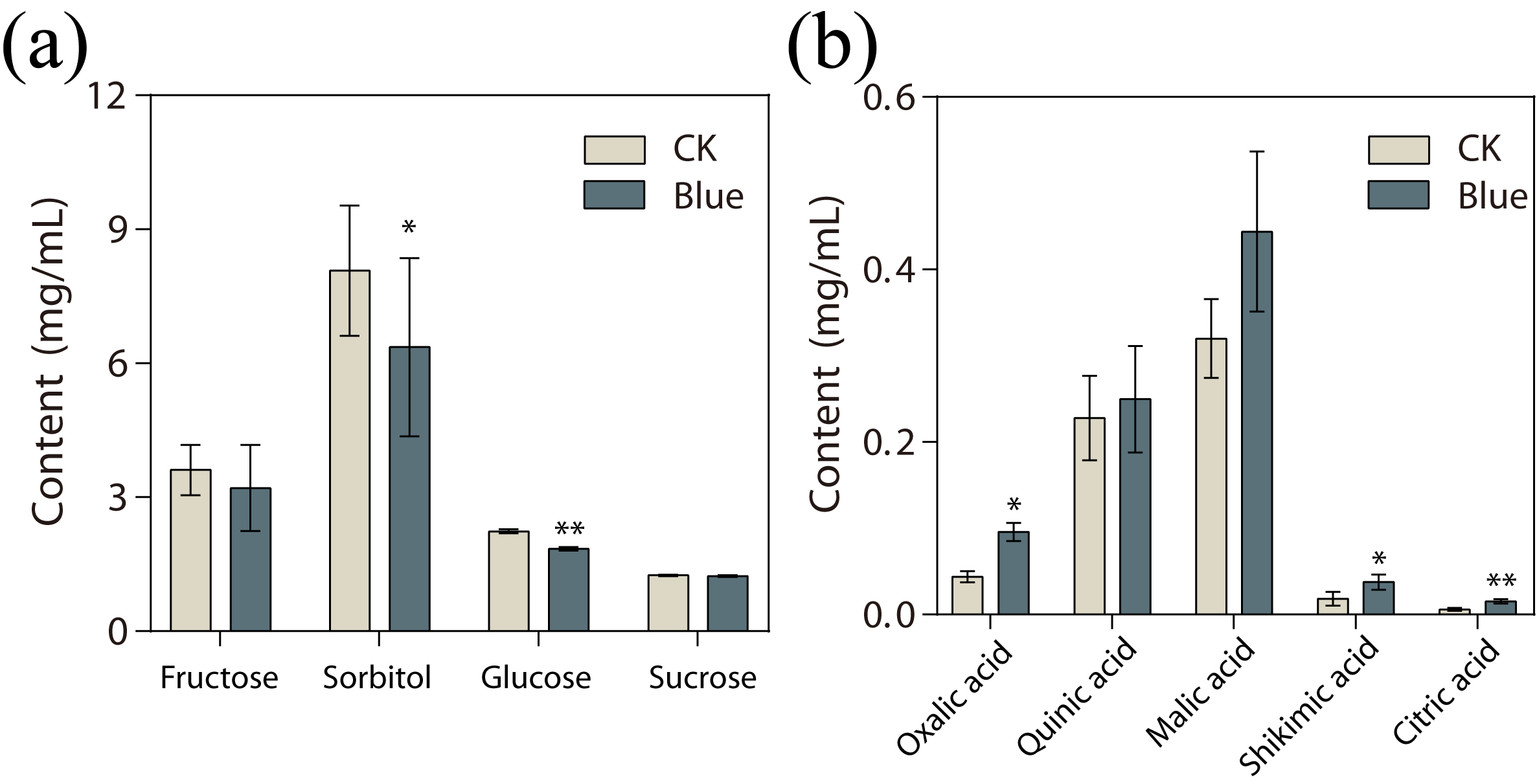
**

**Figure S1. Physiological changes of pear fruit after blue light treatment.**

Contents of plant hormones (a), soluble sugars (b), and organic acids (c) of ‘Cuiguan’ fruits under blue light irradiation. Three biological replicates were performed. Student’s *t*-test was used for statistical analyses; Mean±SD (**P* < 0.05, ***P* < 0.01)

**
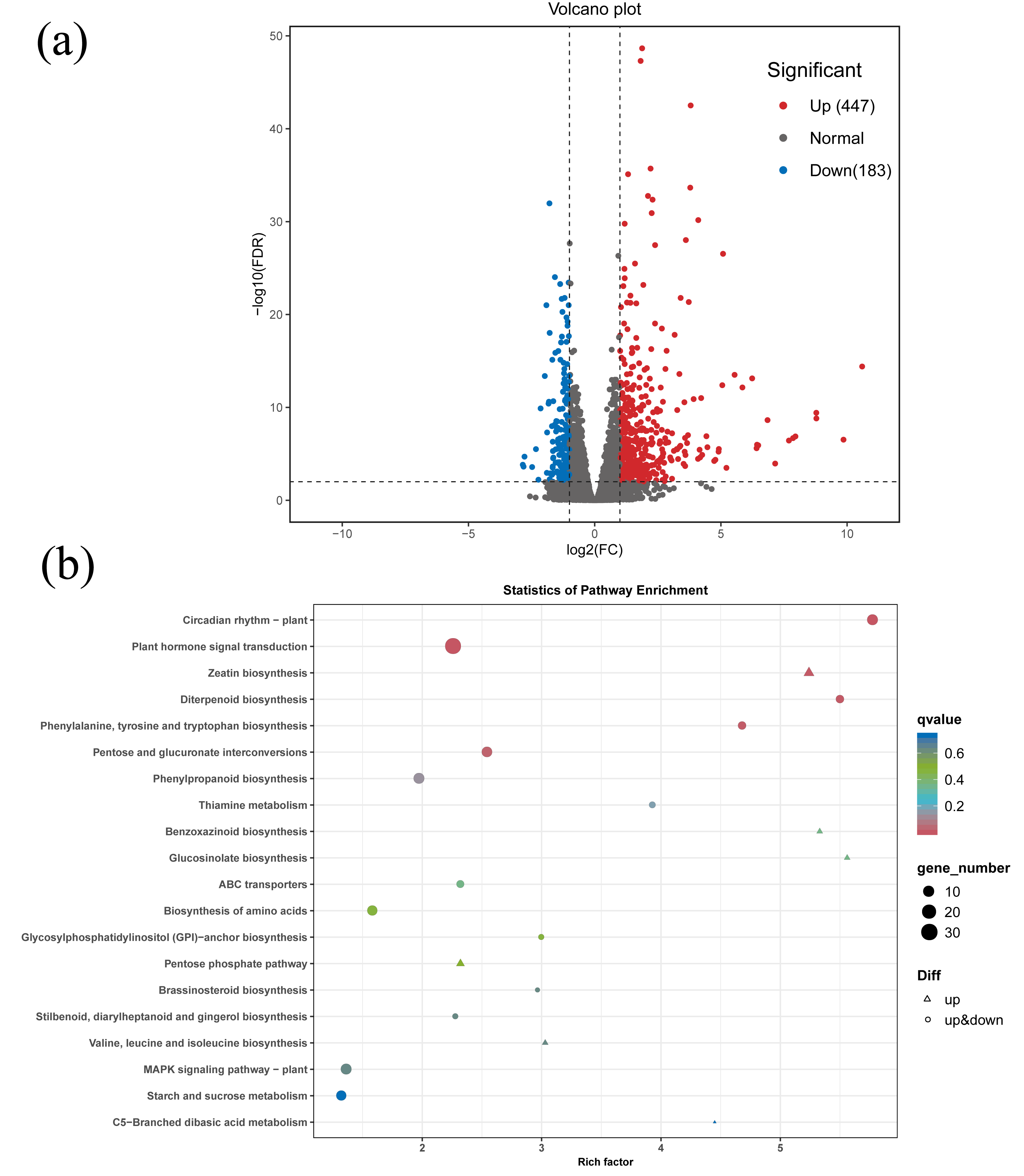
**

**Figure S2 Statistical and enrichment analysis of differentially expressed genes.**

1. Volcano plot of differentially expressed genes.
2. KEGG enrichment of differentially expressed genes.

**
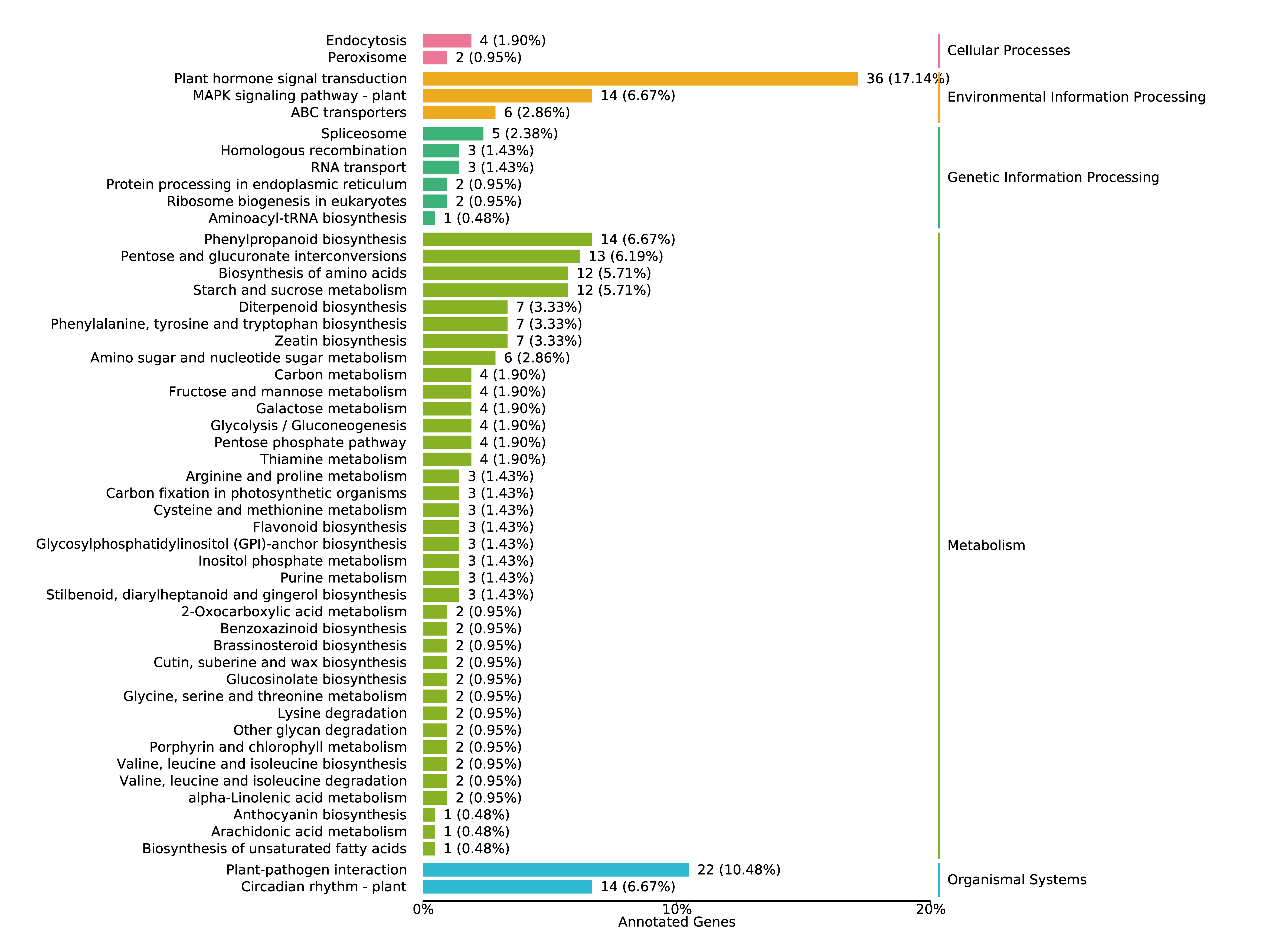
**

**Figure S3 KEGG classification analysis of differentially expressed genes.**

**
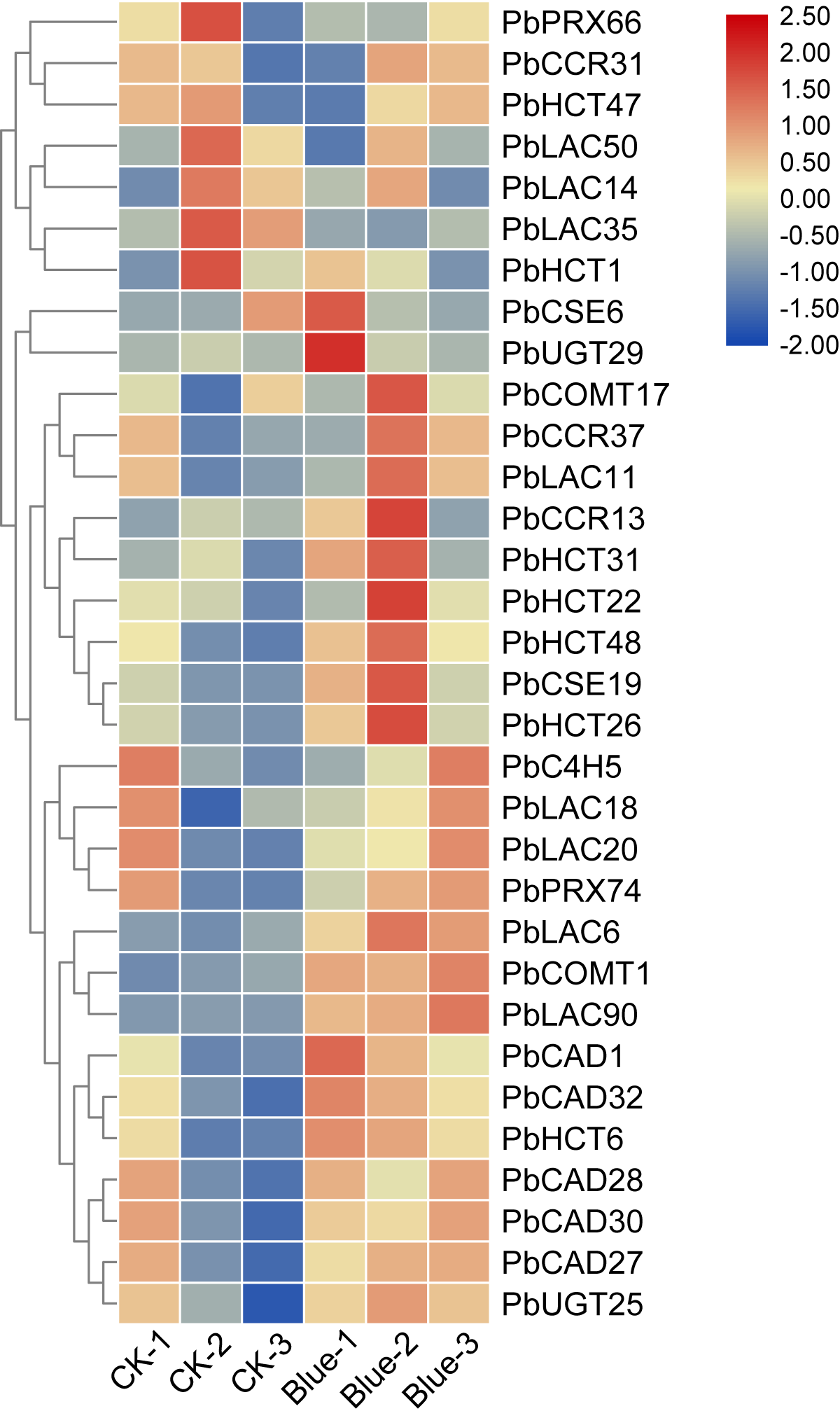
**

**Figure S4 Heatmap cluster analysis of lignin biosyhthetic genes in pear fruits after blue light treatment.**

**
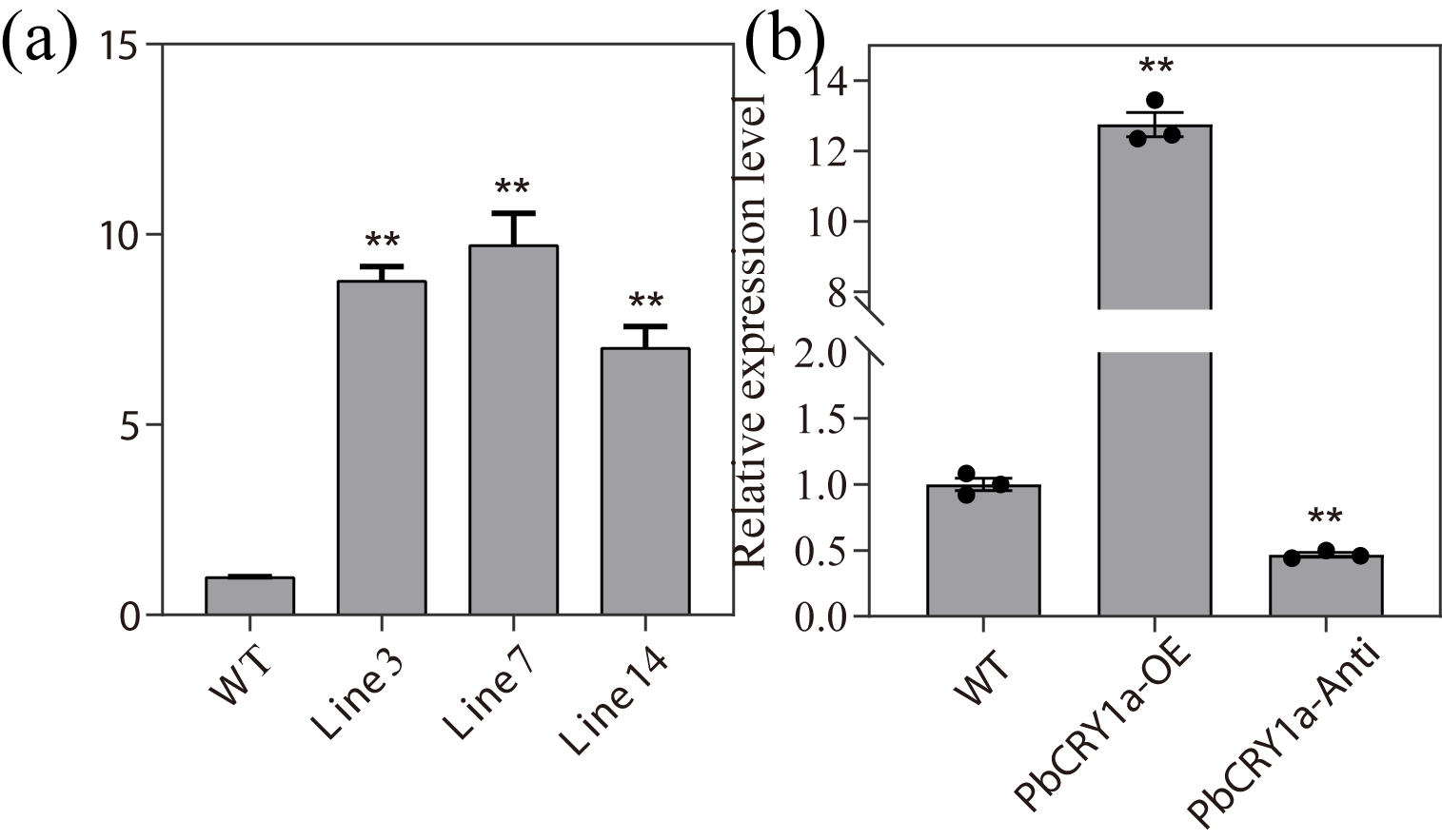
**

**Figure S5.** qRT-PCR verification of gene overexpression and repression (a) qRT-PCR analysis showed the overexpression of PbCRY1a in representative overexpression Arabidopsis lines. (b) qRT-PCR confirmed that PbCRY1a was successfully overexpressed at the PbCRY1a-OE lines, and repressed at PbCRY1a-Anti lines. The ordinate was the mean ± SD of the three biological replicates, and asterisk indicates the significance difference of the students-*t*-test (***p* < 0.01).


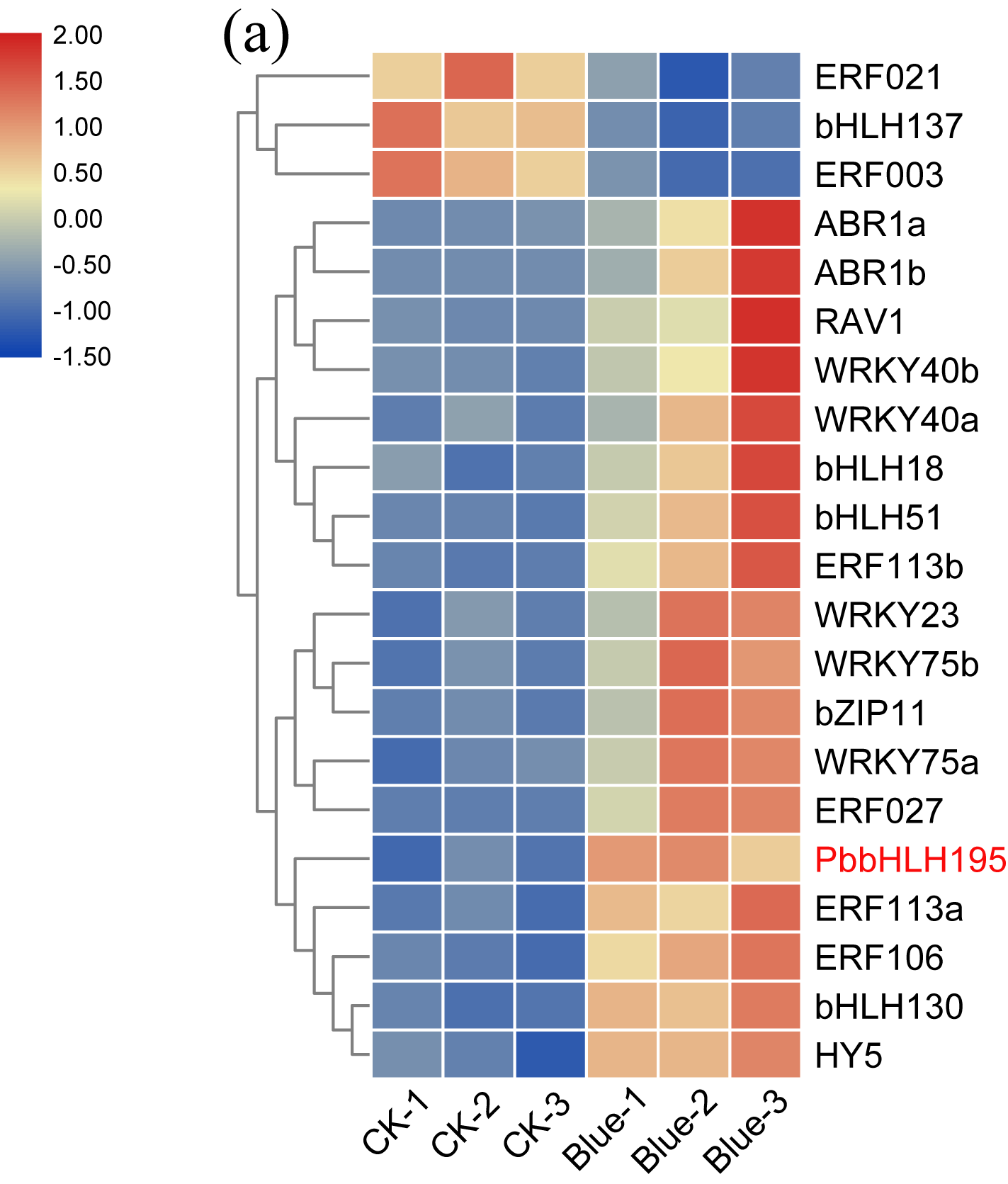


**Figure S6** Heatmap cluster indicates the differentially expressed TFs of pear fruits under blue light based of RNA-sequencing data.

#
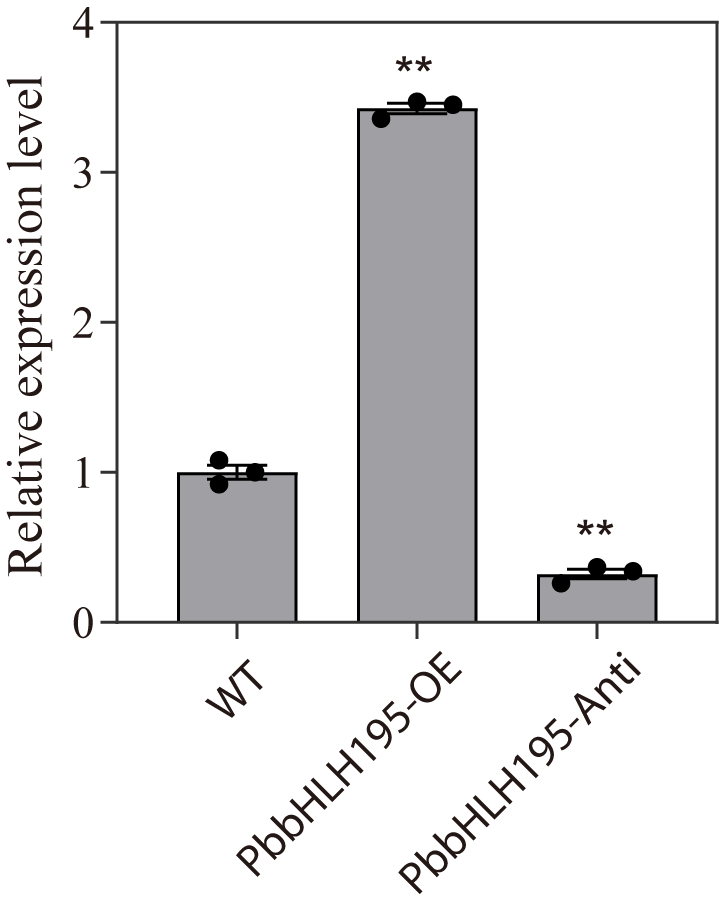


**Figure S7.** qRT-PCR confirmed that PbbHLH195 was successfully overexpressed at the PbbHLH195-OE lines, and repressed at PbbHLH195-Anti lines. The ordinate was the mean ± SD of the three biological replicates, and asterisk indicates the significance difference of the students-*t*-test (***p* < 0.01).


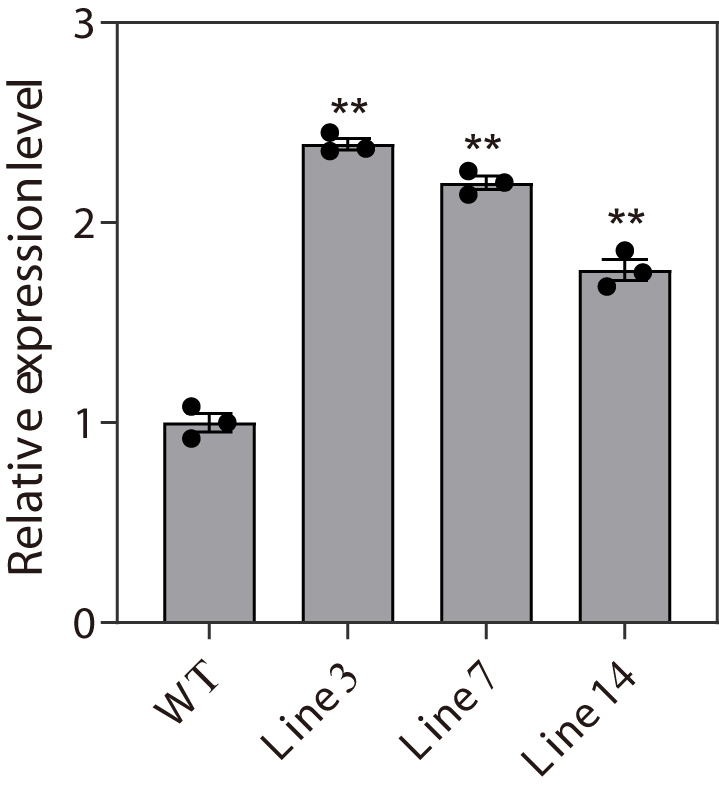


**Figure S8.** The relative expression of *AtSND1* (homology of *PbNSC*) was significantly induced by overexpression PbbHLH195. The ordinate was the mean ± SD of the three biological replicates, and asterisks indicates the significance difference of the students-*t*-test (***p* < 0.01).
